# The effect of cold atmospheric plasma (NO) alone and in combination with NPH insulin on the full-thickness excisional wound healing in a diabetic rat model

**DOI:** 10.17221/109/2022-VETMED

**Published:** 2023-04-28

**Authors:** Ali Curukoglu, Gul Ciray Akbas Gungor, Gokce Akan, Aysel Kukner, Gozde Ogutcu, Melis Kalayci, Meliha Temizel, Fatma Eser Ozgencil

**Affiliations:** ^1^Surgery Department, Faculty of Veterinary Medicine, Near East University, Yakin Dogu St, Nicosia, Mersin, Turkiye; ^2^DESAM Institute, Near East University, Yakin Dogu St, Nicosia, Mersin, Turkiye; ^3^Histology Department, Faculty of Medicine, Near East University, Yakin Dogu St, Nicosia, Mersin, Turkiye; ^4^Experimental Animal Research Center, Faculty of Veterinary Medicine, Near East University, Yakin Dogu St, Nicosia, Mersin, Turkiye

**Keywords:** diabetic wound healing, insulin ointment, nitric oxide

## Abstract

This study was planned to investigate an alternative treatment modality in diabetic wound healing. In this experimental study, the efficacy of both cold atmospheric plasma/nitric oxide (NO) and NPH insulin ointment, recently known to have beneficial effects on wound healing, was investigated in diabetic wound healing. Twenty-four (24) diabetic rats were divided into four groups DC, DI, DNO and DINO (diabetic control, diabetic insulin, diabetic nitric oxide, diabetic insulin + nitric oxide groups). No treatment was applied to the DC group, NPH insulin was applied to the DI group, CAP/NO was applied to the DNO group, and CAP/NO + NPH insulin was applied to the DINO group once daily for 14 days. The wound area reduction and the wound contraction rate were calculated on the basis of the tissue sections taken, and histopathological and genetic analyses were carried out. Compared to the control group, exogenous NO gas was found to be a potent antibacterial agent in the diabetic wound healing, causing a reduction in the wound area (*P* = 0.034), an increased contraction rate (*P* = 0.021), epithelialisation (*P* = 0.02), collagen organisation (*P* = 0.006) and a reduction in the number of inflammatory cells (*P* = 0.002). A significant increase in the expression of IL-8 mRNA was observed (*P* = 0.026). It was concluded that NPH insulin alone contributes to wound healing, but it is not necessary to use it together with exogenous NO gas.

Normal wound healing consists of a series of stages – haemostasis, inflammation, proliferation and remodelling – which occur immediately after injury until complete epithelialisation. Hyperglycaemia in diabetic wounds, where microangiopathy and neuropathy develop, prevents cell proliferation and collagen organisation. The prevention of cell proliferation and collagen formation leads to a decrease in growth factors for fibroblastic and angiogenic activity, inhibits the chemotaxis and phagocytotic activity, ultimately leading to delayed wound healing and increased susceptibility to bacterial infection (Richmond et al. 2013; Powers et al. 2016; Grada et al. 2018). Nitric oxide (NO) is an endogenous gasotransmitter that plays a central role in wound healing and has recently become a popular wound treatment as an endogenous regulator of inflammation and a non-resistant antibacterial agent (Forstermann and Sessa 2012; Malone-Povolny et al. 2019; Bilir 2022). While macrophages, keratinocytes and fibroblasts express high levels of nitric oxide synthase 2 in healthy wounds, its expression is suppressed in diabetic wounds. The NOS (nitric oxide synthase) isoenzymes, both NOS3 (nitric oxide synthase 3) and NOS2 (nitric oxide synthase 2), play an important role in the wound healing process. Moderate levels of NO produced by NOS3 regulate the inflammation responsible for chronic wound healing, and high levels of NO produced by NOS2 act to destroy pathogens that cause acute infections. Cutaneous wound healing has been shown to be severely impaired in the absence of NOS2. Fibroblasts express lower levels of NOS2 and NOS3 in diabetic wounds, and fibroblasts are unable to produce collagen, which prevents them from forming the extracellular matrix (ECM), resulting in decreased wound resistance. In addition, insufficient NO prevents the migration and movement of cell types during the wound healing process. NO modulation has recently been considered as a solution to impaired wound healing. NO is not only an endogenous regulator of inflammation, but also an antibacterial agent that lacks resistance through a combination of nitrosative and oxidative mechanisms. The purpose of both endogenous and exogenous therapeutic NO dosing is to regulate inflammation and tissue remodelling in chronic wounds (Kitano et al. 2017). In this sense, studies on both exogenous NO application in diabetic wounds and the placement of long-term NO providing chitosan hydrogels and polymeric nanoparticles in the wound bed are among the current applications to accelerate wound healing. The use of NO-releasing chitosan hydrogels to increase the efficacy of placenta-produced mesenchymal stem cells in the treatment of ischaemic hind limb wounds in humans has shown promising results and has enhanced angiogenesis (Gronbach et al. 2020; Ahmed et al. 2022). It has been reported that NO contributes to the carbohydrate metabolism and its bioavailability decreases during the development of type 2 diabetes mellitus (T2DM), and that NO donors can improve the insulin signalling, glucose homeostasis and insulin resistance (IR) in T2DM (Ozaydin et al. 2018; Wang and Xu 2020). Topical notr protamine hagedorn (NPH) insulin is widely used in both diabetic and non-diabetic wounds due to its relatively low cost and potential wound healing ability. The use of insulin in topical bioadhesives, hydrogels, local injections, sprays, creams and controlled release of bioactive insulin has been found to reduce the wound healing time (Zhao et al. 2017; Ozaydin et al. 2018; Yu et al. 2019; Wang and Xu 2020).

The aim of the study was the effect of exogenous NO gas and/or NPH insulin ointment applications on wound healing in diabetic rats for 14 days was researched. The effect of reducing the wound area was analysed by clinical, histological and gene expression analyses.

## MATERIAL AND METHODS

### Study design and animals

A total of 24 male Wistar albino rats aged 6 months and weighing 250 g were used for the study. The groups were randomly divided into four equal groups which included: Group 1 – diabetic control group (DC), group 2 – diabetic NPH insulin group (DI), group 3 – diabetic CAP/NO group (DNO), group 4 – diabetic cold atmosphere NO + NPH insulin group (DINO). The duration of the study was 14 days, with treatment applications performed once a day. The study received ethical approval from the Near East University’s Animal Experiments Local Ethics Committee (Reference No.: 2021/141).

### Diabetes model

Diabetes was induced in the rats by a modification of the technique proposed by Saad et al. (1992) with a single dose of 50 mg/kg streptozotocin (STZ) dissolved at pH 6 in a 0.07 M citrate buffer and administered intraperitoneally (Sigma, St. Louis, MO, USA). The rats were fasted for 12 h before and 4 h after the STZ administration, and 0.1 ml of blood was drawn from the tail vein of all the animals on day 1, day 2 and day 14, and the blood glucose levels were examined with a digital glucose meter (Gluco Leader; HMD BioMedical Inc., Hsinchu, Taiwan). On the first day, the drinking water of all the subjects consisted of 15% dextrose, and, after the STZ injection, they were classified as diabetic if their blood glucose level was above 200 mg/dl (11.1 mmol/l) (Riedl et al. 2011).

### Anaesthesia and wound model

The animals were anaesthetised intraperitoneally with 100 mg/kg ketamine hydrochloride (Ketamine; Dutch Farm International, Nederhorst Den Berg, Holland) and 10 mg/kg xylazine hydrochloride (Vetaxyl^®^; Russia). Before creating the wound model, 1 ml/kg meloxicam (Meloxicam^®^; Bavet, Istanbul, Turkiye) was injected subcutaneously. Considering that small wounds spontaneously shrink with the contraction phase of the panniculus muscle in rats, a 3 cm diameter excision wound was created by the same surgeon in the right lateral dorsal region, covering the epidermis, dermis and panniculus carnosus (Dorsett-Martin 2004).

### Wound area reduction and wound contraction rate measurements

Photographs taken by the same researcher on the day of the wound formation, and on the 3^rd^, 7^th^ and 14^th^ day were transferred to ImageJ 1.53k freeware (National Institutes of Health, Rockville, MD, USA) and the wound areas were recorded in cm^2^. The wound area reduction rate between days 0–3, 0–7 and 0–14 were calculated and comparisons were made between the groups (Bae et al. 2012; Anuk et al. 2016):

$$\begin{array}{l} {}[(\text{Created wound area}–\text{measured }\\ \text{wound area})/\text{created wound area}]\times 100 \end{array}$$ (1)

### NPH insulin ointment preparation and application

The NPH insulin ointment was prepared according to Ozaydin et al. (2018). It was applied to the wound area once daily in a 2 mm thin layer in the DI group and in the DINO group and massaged gently until it was absorbed by the wound. In the DINO group, this was undertaken after the application of CAP.

### CAP/NO application

In this study, we used the Cold Atmospheric Nitric Oxide (CAP/NO) device (Inosante Medical Inc., Antalya, Turkiye), a signalling molecule designed to deliver a gas mixture containing NO to biological tissues in veterinary medicine. The device produces exogenous nitrogen monoxide that acts on all the organs and tissues without the need for cell receptors. This CAP/NO application, designed to deliver a gas mixture containing NO to biological tissues, was administered at a dose of 200 ppm once daily for 90 s at a distance of 2 mm with spiralling movements from the periphery of the wounds to the centre and, in the DNO and DINO groups, from the centre to the periphery. Tissue samples for histopathological evaluation were collected from rats under general anaesthesia. Thereafter the rats were sacrificed.

### Histopathological evaluations

Skin samples were taken with surgical scissors to cover the wound area and 0.5 cm of the surrounding healthy tissue and fixed with 10% formaldehyde. After a fixation period of 24–48 h, the tissues were passed through graded alcohol series and xylol and were embedded in paraffin. Microtome (Leica Biosystems, Nubloch, Gemany) serial sections of 4–5 μm thickness were taken from the prepared paraffin blocks. The sections were used to assess the inflammation, angiogenesis, collagen organisation and epithelial regeneration according to the method of Rahman et al. (2019).

### Gene expression analysis

#### TOTAL RNA EXTRACTION AND QUANTIFICATION

Approximately 30 mg of the wound tissue was collected on the first day of the healing procedure prior to treatment and on the 14^th^ day of the treatment. All the tissue samples were then immediately frozen in liquid nitrogen and stored at −80 °C prior to the total RNA preparation. The total RNA from the wound tissues was extracted with a Trizol^™^ Reagent (Invitrogen, Carlsbad, CA, USA) according to the manufacturer’s instructions. The RNA quantity was determined by a NanoDrop^™^ 1000 Spectrophotometer (Thermo Scientific, Wilmington, DE, USA). Afterwards, 1 μg of RNA was reverse transcribed in 20 μl total volume using OligodT and Random Hexamer as the primers using a Revert Aid First Strand cDNA Synthesis Kit (Fermentas Canada Inc., Burlington, ON, Canada).

#### GENE EXPRESSION ANALYSIS BY QUANTITATIVE REAL-TIME PCR (qRT-PCR)

The expression levels of *IL-8*, *VEGFA*, *TGF-β1* and *NOS2* were detected by Real-Time Quantitative Reverse Transcription Polymerase Chain Reaction (qRT-PCR) using a SensiFAST^™^ SYBR No-ROX kit and the Real-Time PCR system (Roche, Mannheim, Germany). Ten-fold dilutions of cDNA synthesised from the total RNA were used. All the samples were amplified in duplicate and the mean values were obtained for further calculations. Primers were designed using the software Universal Probe Library and the primers were synthesised by Integrated DNA Technologies (Skokie, USA).

### Data analysis

A statistical analysis was performed using the SPSS software (Statistical Package for the Social Sciences v25.0; SPSS Inc., Chicago, IL, USA). The quantitative variables were expressed as the mean ± standard deviation (SD), and the qualitative variables were expressed as percentages. The gene expression data were obtained as cycle threshold (Ct) values (Ct = cycle number at which the logarithmic PCR plots cross a calculated threshold line). The expression of each gene was compared between groups using the 2^ΔΔCt^ method (ΔΔCt = Ct of the target gene-Ct of the housekeeping gene). To evaluate the differences between the groups regarding the results of the wound closure rate, the contraction rate and histopathologic evaluation were statistically analysed using the Kruskal-Wallis test [non-parametric analysis of variance (ANOVA)] with a Conover post-hoc analysis. The correlations analysis between the studied gene expression levels were evaluated with the Spearman rank correlation test. Statistical significance was taken as *P* <* *0.05.

## RESULTS

### Macroscopic-clinical findings

**Figure 1:**
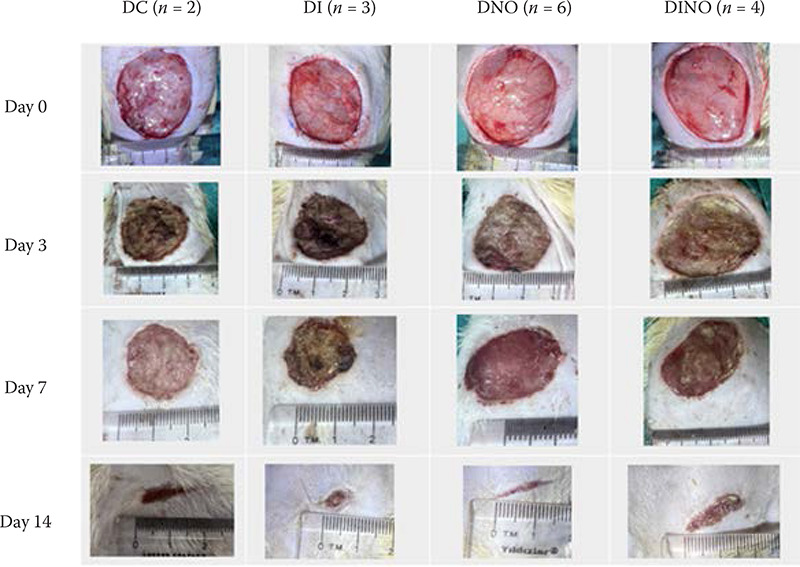
Wound area reduction rates between the groups according to clinical observations DC = diabetic control; DI = diabetic insulin; DINO = diabetic insulin + nitric oxide; DNO = diabetic nitric oxide

**Figure 2:**
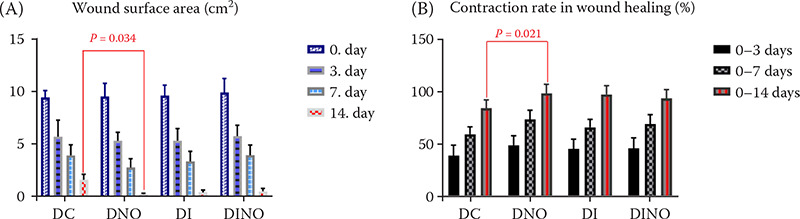
A significant difference was found in the DNO group in terms of both the wound surface area (A) and contraction rate (B) DC = diabetic control; DI = diabetic insulin; DINO = diabetic insulin + nitric oxide; DNO = diabetic nitric oxide

The wounds were the same size in all the study groups at the beginning of treatment (0^th^ day). On day 3, the DC group was the only group in which no wound contraction was observed, while, on day 5, purulent discharge and an uneven appearance of the wound surface were observed in the DC, DI and DINO groups, but no wound discharge was observed in the DNO group. After day 7 post-treatment, an acceleration of the shrinkage of the wound surface was observed in all the groups except the DC group. In the period between the 7^th^ and 12^th^ day, it was observed that all the discharge in the DI and DINO groups had disappeared and the rate of shrinkage of the wound had increased, reaching a healing status in the DNO group. On the 14^th^ day, unlike the control group, it was observed that most of the subjects in the DNO (diabetic nitric oxide) group had their wounds closed and hair regrowth had begun, followed by the DI (diabetic insulin) and DINO (diabetic insulin + nitric oxide) groups (Figure 1). Alternatively, it was observed that the wound area in the DC (diabetic control) group did not shrink proportionally to the presence of the gelatinous biofilms and discharge, which are indicators of infection, on the 3^rd^ and 7^th^ days in the DC (diabetic control) group. In terms of the wound surface area and wound contraction rate, a significant difference was observed between day 0 and day 14 only in the DNO (diabetic nitric oxide) group, both in terms of the wound area reduction (*P* =* *0.034) and contraction rate (*P* = 0.021) (Figure 2A,B).

### Histopathological findings

**Figure 3:**
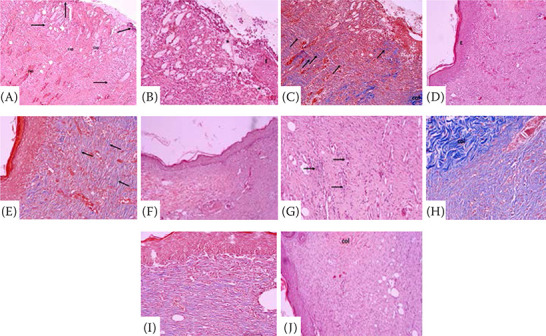
Intergroup evaluation in terms of inflammation, epithelisation, and collagenisation (A) Non-developing epithelialisation in the DC group, capillary perpendicular to the surface (cap), and inflammatory cells (arrows). Haematoxylin staining × 10. (B) Inflammatory cells composed largely of neutrophils. Epithelium (E), oedematous areas (*). Haematoxylin-eosin staining × 20. (C) Increased fibroblastic activity between the congested capillaries in the DC group, disorganised blue dyed collagen fibres (arrows), and collagen fibre bundles (col) in the intact dermis. Masson trichrome staining × 10. (D) In the DI group, the epithelium (E) is better organised than the DC group, capillaries in the dermis layer and the presence of reduced neutrophil cells are observed. Haematoxylin-eosin staining × 10. (E) In the DI group, increased fibroblastic activity, blue dyed collagen bundles that have begun to form in the subepithelial region (*), and thin collagen fibres (arrows) in the inner region are observed. Masson Trichrome staining × 20. (F) It can be seen that epithelialisation occurs in more areas in the NO group than in the DI and DINO groups. Haematoxylin-eosin staining × 10. (G) In the NO group, there are many capillaries that are vertical to the surface and do not contain congestion. It is noteworthy that the neutrophils decreased and the lymphocytes increased in places (arrows). Haematoxylin-eosin staining × 20. (H) In the NO group, denser collagen fibres and bundling were observed in the dermis compared to the DI and DINO groups, and collagen bundles (col) were observed in the intact dermis × 20. (I) In the DINO group, epithelial organisation was observed, and the capillary congestion and inflammation decreased. Collagen bundles (col) are observed in the intact dermis area. Haematoxylin staining × 10. (J) In the DINO group, it can be seen that the collagen fibres are not fully organised and there are fine-diameter fibres. Masson Trichrome staining × 20 DC = diabetic control; DI = diabetic insulin; DINO = diabetic insulin + nitric oxide; DNO = diabetic nitric oxide

Epithelialisation was observed in the wound area of the individuals belonging to the DC group at a rate of 50% less when compared to the other study groups, and, in some cases, it did not occur at all. In the DC group, there was increased activation of fibroblast cells under the epidermis, thin disorganised collagen fibres, non-bundling, prominent congestion, many capillaries perpendicular to the surface, inflammation and oedema formed mainly by the neutrophils (Figure 3A–C). In the DI group, the epithelium was better organised than in the DC group, the presence of increased capillaries in the dermis layer, fewer neutrophils, increased fibroblastic activity, collagen bundles had started to form in the subepithelial region and thin collagen fibres were observed in the inner region (Figure 3D,E). In the DNO group, epithelialisation covering a large part of the wound area, capillaries running perpendicular to the surface and a decreased neutrophil count were observed, and compared to the DI and DINO groups, collagen fibres in the dermis were observed to be denser and bundling began (Figure 3F–H). It was noted that the epithelial organisation in the DINO group was moderate (over 50%), the capillary congestion was reduced, and collagen organisation was similar to the insulin group. Low epithelialisation and increased inflammation were observed in some subjects (Figure 3I,J).

When statistically evaluated in terms of the epithelial regeneration, the number of inflammatory cells, angiogenesis-congestion, and fibroblast activation-collagen fibre organisation. The average value of the epithelial organisation was found to be significantly lower in the DC group than in the DNO group (*P* = 0.02). While the mean number of inflammatory cells was found to be lowest in the DNO group and highest in the DC group, the mean value of the DI and DINO groups was found to be significantly higher than the DNO group (*P* = 0.002). The mean of collagen organisation was found to be significantly lower in the DC group than in the other three groups (*P* = 0.006). There was no significant difference between the four groups in terms of the mean angiogenesis (Table 1).

**Table 1:** Descriptive statistics of the measurements by groups

Parameter	Group	*N*	Mean	SD	P*
Epithelial organisation	DC	6	0.50^a^	0.548	**0.020**
	DI	6	1.33^ab^	1.033	
	DNO	6	2.00^b^	0.632	
	DINO	6	1.50^ab^	0.548	

Number of inflammatory cells	DC	6	3.00^c^	0.000	**0.002**
	DI	6	2.17^b^	0.753	
	DNO	6	1.33^a^	0.516	
	DINO	6	2.17^b^	0.408	

Angiogenesis	DC	6	2.67	0.516	0.867
	DI	6	2.83	0.408	
	DNO	6	2.83	0.408	
	DINO	6	2.83	0.408	

Collagen organisation	DC	6	0.33^a^	0.516	**0.006**
	DI	6	1.50^b^	0.548	
	DNO	6	1.83^b^	0.408	
	DINO	6	1.50^b^	0.548	

*The data were analysed by using the Kruskal-Wallis test (non-parametric ANOVA) with a Conover post-hoc analysis Values in the table are points

DC = diabetic control; DI = diabetic insulin; DINO = diabetic insulin + nitric oxide; DNO = diabetic nitric oxide

### Gene expression analysis in the wound tissues

**Figure 4:**
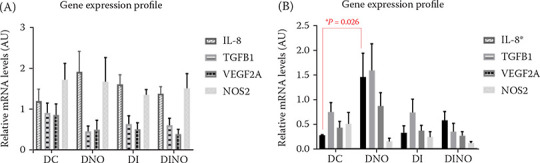
First day (A) and 14^th^ day (B) mRNA expression levels in all the groups DC = diabetic control; DI = diabetic insulin; DINO = diabetic insulin + nitric oxide; DNO = diabetic nitric oxide

The expression levels of *IL-8*, *VEGFA*, *TGF-β1* and *NOS2* were examined on the 1^st^ day before starting the healing procedure and the 14^th^ day of the healing procedure in all the rats from all the treatment groups (Figure 4). The mRNA expression levels were similar between the tissues collected from all the study groups on the first day of the healing procedure before the treatment. The *IL-8* mRNA expression levels were significantly upregulated in the wound tissues collected on day 14 of the treatment from the rats in the NO (nitric oxide) group compared to the wound tissues collected on day 14 of the treatment from the rats in the DC (diabetic control) group (*P* = 0.026). In addition, there was a 2-fold increase in the mRNA expression levels of *VEGFA* and *TGF-β1*, while the mRNA expression levels of the *NOS2* gene decreased 3-fold in the wound tissues of the rats in the NO group compared with the rats in the DC group, but these differences were not significant.

The correlation analyses between the expression levels of the investigated genes were analysed using the Spearman correlation test. The mRNA expression levels of *VEGFA* in wound tissues collected on day 14 were positively correlated with the mRNA expression levels of *TGF-β1* (*r* = 0.713, *P* = 0.001).

Among all the groups, the *IL-8* mRNA was observed to be significantly up-regulated in the wound tissues collected on the 14^th^ day of treatment in the NO group rats compared to the wound tissues collected on the 14^th^ day of treatment in the DC group rats (*P* = 0.026). The data were analysed by using the Kruskal-Wallis test (non-parametric ANOVA).

## DISCUSSION

Diabetic wounds are chronic wounds in which wound healing is impaired at any stage by the effects of hyperglycaemia. The hyperglycaemic environment promotes biofilm formation, making wound healing more difficult (Burgess et al. 2021). In this study, the application of a CAP/NO device at a dose of 200 ppm for 90 s and from a distance of 2 mm significantly contributed to a reduction in the wound area. According to our clinical observations, the wound shrinkage was attributed to the positive effect of the insulin in all the groups except the control group from day 3; NO and NPH separately. On day 5, a purulent discharge, an uneven wound surface and a biofilm in the wound were observed in the DC, DI, DINO groups, but not the DNO group, confirming that NO alone is a potent antibacterial agent. After the 7^th ^day, it was observed that the shrinkage of the wound area accelerated in all the groups except the DC group, and on day 12, the wound discharge disappeared in the DI and DINO groups, and both groups approached the DNO group in terms of the rate of shrinkage of the wound area. On day 14, it was observed that the wounds closed in most of the subjects in the DNO group, and the NO group was followed by the insulin group in terms of the rate of shrinkage of the wound area. In the study, the presence of a wound discharge in the DI group led us to conclude that insulin alone had no antimicrobial activity. It was concluded that the use of exogenous NO gas, which is known to be an antibacterial agent, together with NPH insulin ointment was not clinically useful. The presence of gelatinous biofilms and discharge, a major complication of diabetes in the DC group, had a proportionally negative effect on reducing the wound area, and the subjective antibacterial efficacy of NO alone was demonstrated.

Hyperglycaemia in diabetes causes the formation of ROS (reactive oxygen species) and RNS (reactive nitrogen species) in the blood. While they contribute to healing under normal conditions, when the ROS/RNS levels exceed the antioxidant capacity of a tissue, they prevent the transition of the wound from the inflammatory stage to the proliferative stage (Bansal et al. 2012; Cobos Campos et al. 2013). Reported NO demand in diabetic wounds is gaseous directly to the wound as the primary therapeutic agent, and the treatment dose and duration are controlled by manipulating the concentration and/or flow rate of the therapeutic gas (Shekhter et al. 2005; Ahmed et al. 2022). The exogenous NO gas produced by the “Plason” air plasma device was developed for this purpose. In a previous study, it was applied to both aseptic and purulent 300 mm^2^ wounds in rats, at a daily dose of 500 ppm for 60 s and resulted in healed skin wounds and a reduction in the healing time of about one-third. In the same study, NO was used at concentrations up to 500 ppm once daily for 60 s for 6 days to reduce any toxicity concerns, and tissue hypoxia and a reduction in microbial infection were reported along with increased angiogenesis in both the infected and clean wounds (Shekhter et al. 2005). In another study supporting this study, the gas NO was found to be safe when used at doses of 5, 25, 75 and 200 ppm for 8 h in a mouse lymphocyte model. The use of NO at concentrations of 5–200 ppm did not damage the ECM and increased the lymphocyte proliferation (Moeen Rezakhanlou et al. 2011). Daily 60-s exposure to the NO-containing gas flow (500 ppm) produced by the “Plason” air-plasma system was found to reduce the healing time in both aseptic and septic 300 mm^2^ wounds.

Similar to the device reported in the study, NO was dosed with a device that produces NO gas from atmospheric air at a dose of 200 ppm for 90 s and from a distance of 2 mm. Photographs taken on day 0, 3, 7 and 14 were evaluated for their reduction in the wound area. As a result, only one difference was found in the DNO group compared to the control group (*P* = 0.034). However, when the wound contraction rate of the groups between 0–3, 0–7 and 0–14 days was evaluated, a difference was found in the DNO group compared to the control group (*P* = 0.021). Our results are consistent with the previous mentioned studies regarding the contribution of exogenous NO dosing to the wound reduction. It was found that the use of NO alone provided the best results in diabetic rat wounds, and it was found that the combined use of NPH insulin with NO gas did not provide a significant benefit.

The use of topical NPH insulin in diabetic wounds involves applying dressings in the form of bioadhesives and hydrogels to the wound area, which has promising results (Zhao et al. 2017; Yu et al. 2019). The priority in the management of diabetic wounds should be to control inflammation, as the persistence of chronic inflammation prevents the wounds from progressing from the inflammatory phase to other phases of wound healing (Miller et al. 2004). In the study, the mean number of inflammatory cells, which is an indicator of chronic inflammation and interruption of wound healing, was lowest in the DNO group among the four groups, and the mean value of the DI and DINO groups was higher than that of the DNO group (*P* = 0.002) and the highest mean value in the DC group, leading us to conclude that NO is important in triggering positive changes in the chronic inflammatory response.

In our study, we found that when NO was applied once daily for 90 s for 6 days, the capillary vessels and fibroblasts decreased and marginal epithelialisation developed on day 14, while immature granulation tissue was observed in the control group. Similarly, the study found that the mean value of epithelial organisation was lower in the DC group compared to the DNO group (*P* = 0.02), leading us to conclude that NO increased the epithelialisation value from day 14. In a previous study in rabbits with severe *Staphylococcus aureus* infected wounds, the application of 200 ppm NO did not affect the re-epithelialisation and angiogenesis, but increased the collagen deposition and wound strength and reduced the bacterial load after three days of treatment by a log (Ghaffari et al. 2007). However, it has also been reported that intermittent NO dosing of up to 500 ppm, administered once daily for 60 s for 6 days, resulted in increased angiogenesis in rats with infected and clean wounds (Shekhter et al. 2005). The fact that no significant difference in the mean angiogenesis was found in all the groups at a 200 ppm for 90 s of NO dose supports the findings of Ghaffari et al. (2007). The fact that the mean collagen build-up in the study was lower in the control group than in the other three groups (*P* = 0.006) suggests that both the NO and insulin administration contributed to the collagen build-up.

Growth factors are endogenous signalling molecules that regulate cellular responses to the wound healing process, and their concentrations are often imbalanced in diabetic wounds as opposed to acute wounds. This leads to a prolonged inflammatory phase and persistent degradation of ECM components (Oztopalan et al. 2017; Malone-Povolny et al. 2019).

*VEGFA* (vascular endothelial growth factor A) plays an important role in the initiation of angiogenesis through proliferation and migration of endothelial progenitor cells (EPCs). In normal wound healing, angiogenesis begins with the emergence of new blood vessels 3 days after injury (Tonnesen et al. 2000; Werner and Grose 2003). In patients with diabetes, hypoxia in the wound microenvironment is the main stimulus for the *VEGFA* uptake into the wound area. This contributes to vascular repair and re-endothelialisation through the secretion of *VEGFA* and supports the blood supply to diabetic foot ulcers as the formation of NO by endothelial cell decreases (Yamakawa and Hayashida 2019; Ahmed et al. 2022; Park et al. 2022).

*TGF-β* (transforming growth factor β) is an effective growth factor for wound healing by regulating cell proliferation, migration, ECM production and immune modulation. *TGF-β1* mediates fibrosis in wounds, while *TGF-β3* serves to promote scarless healing. *TGF-β1* is released in the acute response to injury, and this immediate release causes the migration of macrophages and fibroblasts to the wound (Lichtman et al. 2016). *TGF-β* has also been reported to trigger collagen synthesis and angiogenesis, increase keratinocyte migration by binding to specific cell surface receptors, and greatly facilitate re-epithelialisation (Peplow and Chatterjee 2013; Lichtman et al. 2016; Oztopalan et al. 2017). However, *TGF-β1* has been found to be a determinant of epithelialisation rather than keratinocyte migration and proliferation and is found at high levels in wound healing after the onset of epithelialisation. *TGF-β1* has been reported to interact with *NOS2*, which are central modulators of wound healing, and generally suppresses the *NOS3* expression, and activates NO by inducing latent *TGF-β1* (Mohajeri-Tehrani et al. 2014).

When the mRNA expression levels of the tissue samples of the study group were evaluated, it was found that the mRNA expression levels of *VEGFA* and *TGF-β1* doubled in the wound tissues of the NO groups compared to the DC group rats on the 14^th^ day, but these findings were not found to be statistically significant. The correlation analysis between the expression levels of the studied genes showed that the mRNA expression levels of *VEGFA* and *TGF-β1* had a positive correlation on day 14 (*r* = 0.713, *P* = 0.001). Although not significant in the NO group, the fact that the two factors were high in the NO group and correlated with each other suggested that they contributed to both angiogenesis and epithelialisation.

The NO produced by *NOS2* in wounds increases the *VEGF* expression, contributes to the epithelial regeneration, and improves the blood flow to ischemic tissue as a vasodilator (Marcinkiewicz et al. 1995). The overexpression of *NOS2* has been associated with septic shock, in which a strong inflammatory reaction is observed. Responsible for *NOS2* expression during inflammation, bacterial products, such as lipopolysaccharide (LPS) are M1 macrophages, which are induced by inflammatory cytokines and have an important role in host defence against intracellular pathogens. *In vitro*, mouse macrophages express high levels of *NOS2* in response to many stimuli, including LPS and cytokines (Bilir 2022). It has been stated that hyperglycaemia in diabetes affects the *NOS3* activity and, therefore, topical NO applications from exogenous sources can play an important role (Zahid et al. 2019; Ahmed et al. 2022). This led us to agree with the study that TGF-β1 is essentially a determinant of epithelialisation, which is found at high levels in the wound area during the onset of epithelialisation.

Additionally, it interacts with *NOS2*, which is the central modulator of wound healing, and generally suppresses the *NOS2* expression, and that NO activates by inducing latent *TGF-β* (Marcinkiewicz et al. 1995; Shekhter et al. 2005). It has been stated that dosing of 500 ppm daily NO for 90 s to aseptic and purulent wounds contributes to wound healing by increasing the *NOS1*, *NOS2* and *NOS3* levels. However, in the analyses performed on day 14 in the diabetic rats, it was observed that there was a three-fold decrease in the *NOS2* expression in the DNO group compared to the DC group, although this was not significant. This result seems to support a previous study that *TGF-β1* interacts with *NOS2*, the central modulator of wound healing, which is stated to be found at high levels in wound healing after the onset of epithelialisation, and generally suppresses *NOS2* expression. Likewise, it can be interpreted that the low *NOS2* expression is aimed at reducing inflammation, supporting the study that correlates M1 macrophages responsible for the *NOS2* expression in inflammation with an excessive inflammatory response (Forstermann and Sessa 2012).

*IL-8* (interleukin 8) is a chemotactic factor for fibroblasts, endothelial cells, monocytes, T-cells and neutrophils. Its high levels have been observed in non-healing human burn wounds and have been suggested to be associated with impaired wound repair (Iocono et al. 2000). However, an *in vitro* stimulating effect of *IL-8* on keratinocyte proliferation has been reported. The topical application of this chemokine *in vivo* onto human skin grafts in a chimeric mouse model has been observed to induce re-epithelialisation as a result of the increased keratinocyte proliferation (Werner and Grose 2003). *IL-8* has also been found to be the major bioactive chemoattractant for neutrophils in human blister and skin graft donor site wound fluids (Rennekampff et al. 2000). Expression of *IL-8* increases within 4 h after injury, and *IL-8* mRNA is not detected in foetal wounds until 12 h, whereas expression of this chemokine persists up to 72 h in adult wounds. These results suggest that a decreased inflammatory cytokine response in the foetal tissue may be responsible for the lack of inflammation in foetal wound healing, which may contribute to scarless wound repair (Werner and Grose 2003). In the study, the *IL-8* mRNA expression levels were observed to increase in the tissues taken at the end of the treatment when the NO group was compared with the DC group (*P* = 0.026), and this result led to the conclusion that *NOS2* significantly increased the regulation of *IL-8*, which is a chemotactic factor for many immune cells.

When the clinical, histopathological and gene expression analysis results were evaluated together in the study; the applications of exogenous NO at a dose of 200 ppm for 90 s from a distance of 2 mm in diabetic wound healing exhibited an antibacterial effect, increased wound area reduction, wound contraction rate, epithelialisation and collagen organisation, and modulated inflammation with an increased IL-8 expression were observed in chronic wounds by reducing the number of inflammatory cells, thus, contributing to wound healing.
